# Supplementary material for: Resolving the full spectrum of human genome variation using Linked-Reads
Source: Genome Res. 2019 Apr;29(4):635–45. doi: 10.1101/gr.234443.118 (PMC6442396; doi:10.1101/gr.234443.118)
Supplement: Supplemental Material [file supp_gr.234443.118_Supplemental_longranger-2.2.zip › longranger-2.2.tar.bz2!/lr2.2-public/lib/stan/cmdstan/stan/doc/api/html/unvalued__argument_8hpp_source.html]

Stan: src/stan/gm/arguments/unvalued\_argument.hpp Source File


|  |  |
| --- | --- |
|  | Stan  2.5.0  probability, sampling & optimization |


- Main Page
- Related Pages
- Namespaces
- Classes
- Files

- File List
- File Members

All Classes Namespaces Files Functions Variables Typedefs Enumerations Enumerator Friends Macros Pages

- src
- stan
- gm
- arguments

unvalued\_argument.hpp

Go to the documentation of this file.

1 #ifndef STAN\_\_GM\_\_ARGUMENTS\_\_UNVALUED\_\_ARGUMENT\_\_BETA

2 #define STAN\_\_GM\_\_ARGUMENTS\_\_UNVALUED\_\_ARGUMENT\_\_BETA

3 #include <iostream>

4

5 #include <vector>

6 #include <stan/gm/arguments/argument.hpp>

7

8 namespace stan {

9

10  namespace gm {

11

12  class unvalued\_argument: public argument {

13

14  public:

15

16  unvalued\_argument()

17  : \_is\_present(false) {}

18

19  void print(std::ostream\* s, const int depth, const std::string prefix) {}

20

21  void print\_help(std::ostream\* s, const int depth, const bool recurse = false) {

22  if (!s)

23  return;

24

25  std::string indent(indent\_width \* depth, ' ');

26  std::string subindent(indent\_width, ' ');

27

28  \*s << indent << \_name << std::endl;

29  \*s << indent << subindent << \_description << std::endl;

30  \*s << std::endl;

31

32  }

33

34  bool parse\_args(std::vector<std::string>& args, std::ostream\* out,

35  std::ostream\* err, bool& help\_flag) {

36  if (args.size() == 0)

37  return true;

38

39  if ((args.back() == "help") || (args.back() == "help-all")) {

40  print\_help(out, 0);

41  help\_flag |= true;

42  args.clear();

43  return true;

44  }

45

46  \_is\_present = true;

47  return true;

48  };

49

50  bool is\_present() {

51  return \_is\_present;

52  }

53

54  protected:

55  bool \_is\_present;

56  };

57

58  } // gm

59 } // stan

60 #endif

stan::gm::unvalued\_argument::\_is\_present

bool \_is\_present

**Definition:** unvalued\_argument.hpp:55

stan::gm::unvalued\_argument::parse\_args

bool parse\_args(std::vector< std::string > &args, std::ostream \*out, std::ostream \*err, bool &help\_flag)

**Definition:** unvalued\_argument.hpp:34

stan::gm::unvalued\_argument

**Definition:** unvalued\_argument.hpp:12

stan::gm::unvalued\_argument::is\_present

bool is\_present()

**Definition:** unvalued\_argument.hpp:50

argument.hpp

stan::gm::argument::\_name

std::string \_name

**Definition:** argument.hpp:76

stan::gm::argument::indent\_width

int indent\_width

**Definition:** argument.hpp:79

stan::gm::unvalued\_argument::unvalued\_argument

unvalued\_argument()

**Definition:** unvalued\_argument.hpp:16

stan::gm::unvalued\_argument::print

void print(std::ostream \*s, const int depth, const std::string prefix)

**Definition:** unvalued\_argument.hpp:19

stan::gm::argument

**Definition:** argument.hpp:12

stan::gm::unvalued\_argument::print\_help

void print\_help(std::ostream \*s, const int depth, const bool recurse=false)

**Definition:** unvalued\_argument.hpp:21

stan::gm::argument::\_description

std::string \_description

**Definition:** argument.hpp:77


---

[ Stan Home Page ]

*© 2011–2014,
Stan Development Team.*
